# Transanal minimally invasive surgery (TAMIS) versus endoscopic submucosal dissection (ESD) for resection of non-pedunculated rectal lesions (TRIASSIC study): study protocol of a European multicenter randomised controlled trial

**DOI:** 10.1186/s12876-020-01367-z

**Published:** 2020-07-13

**Authors:** Nik Dekkers, Jurjen J. Boonstra, Leon M. G. Moons, Roel Hompes, Barbara A. Bastiaansen, Jurriaan B. Tuynman, Arjun D. Koch, Bas L. A. M. Weusten, Apollo Pronk, Peter A. Neijenhuis, Marinke Westerterp, Wilbert B. van den Hout, Alexandra M. J. Langers, Jolein van der Kraan, Alaa Alkhalaf, Jonathan Y. L. Lai, Frank ter Borg, Hans Fabry, Eric Halet, Matthijs P. Schwartz, Wouter B. Nagengast, Jan Willem A. Straathof, Rogier W. R. ten Hove, Leendert H. Oterdoom, Christiaan Hoff, Eric J Th Belt, David D. E. Zimmerman, Muhammed Hadithi, Hans Morreau, Erienne M. V. de Cuba, Jeroen W. A. Leijtens, Hans F. A. Vasen, Monique E. van Leerdam, Eelco J. R. de Graaf, Pascal G. Doornebosch, James C. H. Hardwick

**Affiliations:** 1 Department of Gastroenterology & Hepatology, Leiden University Medical Center, Albinusdreef 2, 2333 ZA Leiden, The Netherlands; 2 Department of Gastroenterology & Hepatology, University Medical Center Utrecht, Utrecht, The Netherlands; 3 Department of Surgery, Amsterdam University Medical Center, Amsterdam, The Netherlands; 4 Department of Gastroenterology & Hepatology, Amsterdam University Medical Center, Amsterdam, The Netherlands; 5 Department of Gastroenterology & Hepatology, Erasmus Medical Center, Rotterdam, The Netherlands; 6 Department of Gastroenterology & Hepatology, St. Antonius Hospital, Nieuwegein, The Netherlands; 7 Department of Surgery, Diakonessenhuis, Utrecht, The Netherlands; 8 Department of Surgery, Alrijne hospital, Leiderdorp, The Netherlands; 9 Department of Surgery, Haaglanden Medical Center, The Hague, The Netherlands; 10 Department of Medical Decision Making & Quality of Care, Leiden University Medical Center, Leiden, The Netherlands; 11 Department of Gastroenterology & Hepatology, Isala hospital, Zwolle, The Netherlands; 12 Department of Gastroenterology & Hepatology, Haaglanden Medical Center, The Hague, The Netherlands; 13 Department of Gastroenterology & Hepatology, Deventer Hospital, Deventer, The Netherlands; 14 Department of Surgery, Bravis Hospital, Bergen op Zoom, The Netherlands; 15 Department of Gastroenterology & Hepatology, Bravis Hospital, Bergen op Zoom, The Netherlands; 16 Departmet of Gastroenterology & Hepatology, Meander Medical Center, Amersfoort, The Netherlands; 17 Department of Gastroenterology & Hepatology, University Medical Center Groningen, Groningen, The Netherlands; 18 Department of Gastroenterology & Hepatology, Maastricht University Medical Center, Maastricht, The Netherlands; 19 Department of Gastroenterology & Hepatology, Alrijne Hospital, Leiderdorp, The Netherlands; 20 Department of Gastroenterology & Hepatology, Hagaziekenhuis, The Hague, The Netherlands; 21 Department of Surgery, Medical Center Leeuwarden, Leeuwarden, The Netherlands; 22 Department of Surgery, Albert Schweitzer Hospital, Dordrecht, The Netherlands; 23 Department of Surgery, Elisabeth-TweeSteden Ziekenhuis, Eindhoven, The Netherlands; 24 Department of Gastroenterology & Hepatology, Maasstad Hospital, Rotterdam, The Netherlands; 25 Department of Pathology, Leiden University Medical Center, Leiden, The Netherlands; 26 Pathan B.V. – Pathology Laboratorium, Rotterdam, The Netherlands; 27 Department of Surgery, Laurentius Hospital, Roermond, The Netherlands; 28 Department of Gastroenterology and Hepatology, Netherlands Cancer Institute, Amsterdam, The Netherlands; 29 Department of Surgery, IJsselland Hospital, Capelle aan den IJssel, The Netherlands

**Keywords:** Endoscopic submucosal dissection, Transanal minimally invasive surgery, Rectal cancer, Adenoma

## Abstract

**Background:**

In the recent years two innovative approaches have become available for minimally invasive *en bloc* resections of large non-pedunculated rectal lesions (polyps and early cancers). One is Transanal Minimally Invasive Surgery (TAMIS), the other is Endoscopic Submucosal Dissection (ESD). Both techniques are standard of care, but a direct randomised comparison is lacking. The choice between either of these procedures is dependent on local expertise or availability rather than evidence-based. The European Society for Endoscopy has recommended that a comparison between ESD and local surgical resection is needed to guide decision making for the optimal approach for the removal of large rectal lesions in Western countries. The aim of this study is to directly compare both procedures in a randomised setting with regard to effectiveness, safety and perceived patient burden.

**Methods:**

Multicenter randomised trial in 15 hospitals in the Netherlands. Patients with non-pedunculated lesions > 2 cm, where the bulk of the lesion is below 15 cm from the anal verge, will be randomised between either a TAMIS or an ESD procedure. Lesions judged to be deeply invasive by an expert panel will be excluded. The primary endpoint is the cumulative local recurrence rate at follow-up rectoscopy at 12 months. Secondary endpoints are: 1) Radical (R0-) resection rate; 2) Perceived burden and quality of life; 3) Cost effectiveness at 12 months; 4) Surgical referral rate at 12 months; 5) Complication rate; 6) Local recurrence rate at 6 months. For this non-inferiority trial, the total sample size of 198 is based on an expected local recurrence rate of 3% in the ESD group, 6% in the TAMIS group and considering a difference of less than 6% to be non-inferior.

**Discussion:**

This is the first European randomised controlled trial comparing the effectiveness and safety of TAMIS and ESD for the *en bloc* resection of large non-pedunculated rectal lesions. This is important as the detection rate of these adenomas is expected to further increase with the introduction of colorectal screening programs throughout Europe. This study will therefore support an optimal use of healthcare resources in the future.

**Trial registration:**

Netherlands Trial Register, NL7083, 06 July 2018.

## Background

Colorectal cancer (CRC) has the second highest incidence rate of all cancers in Europe with an annual incidence rate of approximately 500.000 of which 175.000 are located in the rectum [1].. Since the introduction of population based screening, CRCs are more often detected at an earlier disease stage than symptom-detected CRCs [2]. Resection of pre-malignant precursors has shown to lower the mortality rate due to CRC by 50% [3]. Along with the clearly benign polyps and clearly invasive cancers, colorectal cancer screening also detects many lesions in the act of progressing from one to the other. These lesions present a diagnostic challenge and complex clinical decision making to avoid overtreatment with unnecessary mortality and morbidity on the one hand but also undertreatment on the other. This dilemma is most acute for rectal lesions where standard surgical resection techniques are associated with higher rates of mortality and serious morbidity, such as a permanent stoma and sexual dysfunction, which can be avoided by organ sparing techniques [4].

Ideally, preoperative staging would allow for accurate prediction of invasive cancer and the chance of local lymph node metastases. Unfortunately, current preoperative staging is far from perfect. This is true for all available staging modalities (MRI, endoscopic ultrasound, advanced optical endoscopic imaging) both individually and in combination. This has been shown by a previous randomised study of organ sparing treatment of rectal polyps, the TREND study: 13% of the lesions preoperatively staged as benign turned out to be malignant [5]. Currently most lesions that are not overtly cancerous on endoscopic inspection are resected by piecemeal Endoscopic Mucosal Resection (pEMR) in Western countries. However, piecemeal resection has an important disadvantage in that it prevents optimal histological assessment. This can make histological distinction between a benign and a malignant lesion impossible leading to unnecessary surgical resections or to under staging and undertreatment. The safety and feasibility of *en bloc* resections in the rectum, combined with the limitations of preoperative staging are leading to a shift away from pEMR to *en bloc* resection of large lesions in the rectum. Furthermore, a recent cost-effectiveness analysis suggests that an *en bloc* resection strategy might also be cheaper than a piecemeal resection strategy for rectal lesions by reducing the numbers of patients requiring additional radical rectal surgery [6].

Lesions can be removed *en bloc* with a flexible endoscope by Endoscopic Submucosal Dissection (ESD). ESD results in high *en bloc* rates and low recurrence rates of around 2% [7]. However, ESD has longer procedure times, is difficult to perform and associated with relatively high rates of perforation (5%) [8]. Fortunately, the clinical consequences of perforation in the rectum are usually limited and can almost always be treated conservatively. Several surgical techniques are also available for local *en bloc* resection of large non-pedunculated rectal lesions. Such as Transanal Endoscopic Micosurgery (TEM) or Transanal Minimally Invasive Surgery (TAMIS). The TAMIS technique has largely superseded classical TEM since it requires minimal investment in specialised equipment. Here the lesion is removed transanally with the use of a silicon-rubber port and standard laparoscopic instruments. Compared to ESD it also has a relatively short learning curve, short procedure times and is financially well compensated.

No reports have been published comparing TAMIS and ESD directly. Two recently published meta-analysis comparing TAMIS/TEM to ESD concluded a similar rate of adverse events, recurrence rate and *en bloc* resection rate [9, 10]. Regarding the procedure and hospitalization duration both papers came to a different conclusion. The first concluded that ESD was associated with shorter procedure times and duration of hospitalization [10], the second concluded that there was no difference [9]. However, both meta-analyses largely included ESD procedures that were conducted in Asian countries. As a result the findings may not be representative for the daily practice in the West where the results of ESD tend to be inferior. The aim of the TRIASSIC study is to perform a multicentre, randomised controlled study comparing ESD to TAMIS for the *en bloc* removal of large non-pedunculated rectal lesions in a Western population. This is important as the detection rate of these lesions has increased greatly with the introduction of screening programs. This study will enable the optimal use of healthcare resources in the future.

## Methods/design

### Hypothesis

We hypothesise that ESD will lead to non-inferior recurrence rates in lesions that prove to be benign. We hypothesise that TAMIS will have a higher R0 resection rate for lesions that prove to be invasive but that this will not translate to a reduced need for additional surgery. We further hypothesise that ESD will lead to less serious complications than TAMIS and lower societal costs.

### Objective

The primary aim of this study is to compare both procedures with regard to local recurrence rates at 12 months. The secondary aims are to compare costs, complication rates and the burden perceived by patients in both the short and long term between the two procedures.

### Design

**Fig. 1:**
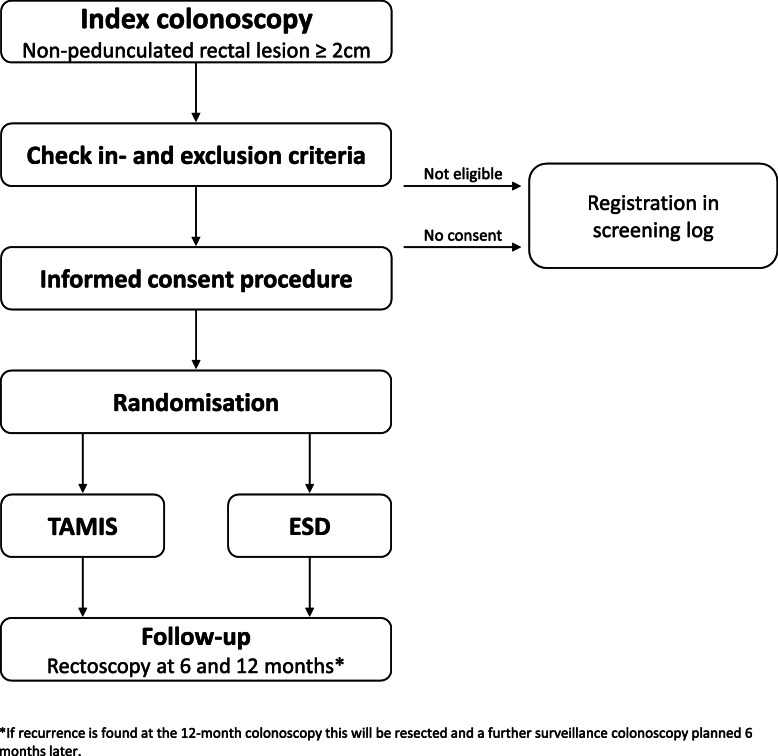
Flowchart of the TRIASSIC study. *Abbreviations:* TAMIS: Transanal Minimally Invasive Surgery, ESD: Endoscopic Submucosal Dissection

This will be a multicentre randomised non-inferiority trial comparing TAMIS and ESD in patients with large rectal lesions. The flowchart of the design of the TRIASSIC study is shown in Fig. 1.

### Randomisation

Patient data will be entered into a cloud-based electronic data capture system (Castor). This system will randomise patients to either the TAMIS or the ESD group. Stratification will take place for the distance of the lesion to the dentate line and lesion size.

### Blinding

Blinding was deemed unfeasible for this trial since both procedures are very different in nature, performed by different specialists and require different associated care facilities within the hospital.

### Study population

In order to be eligible to participate in this study, a subject must meet all of the following **inclusion criteria**:

1. Non-pedunculated lesion > 2 cm in the rectum where the bulk of the lesion is below 15 cm from the anal verge found at endoscopy
2. ≥18 years old
3. Written informed consent

A subject is not eligible for inclusion in case of presence of any of the following **exclusion criteria**:

1. Features of advanced disease or deep-submucosal invasion at optical endoscopic evaluation*
2. Features of advanced disease on cross-sectional imaging*
3. Prior endoscopic resection attempt
4. The risks of treatment are felt to exceed the benefits.

* Where there is discordance in the results, the optical endoscopic evaluation will be given the most weight and the case discussed by an expert panel.

### Participating centres

The TRIASSIC study will be performed in the Netherlands in at least 5 academic and 10 non-academic centres. The following hospitals are participating in the trial: Leiden University Medical Center, Amsterdam University Medical Center (Amsterdam Medical Center and Vrije Universiteit University Medical Center), University Medical Center Utrecht, Erasmus medical Center, IJsselland hospital, Alrijne hospital, Haaglanden Medical Center, Diakonessenhuis, Deventer hospital, Meander Medical Center and the St. Antonius hospital. The following hospitals have shown interest in participation: Bravis hospital, Isala hospital, Albert Schweitzer hospital, Maasstad hospital, Netherlands Cancer institute, University Medical Center Groningen, Maastricht University Medical Center, Medical Center Leeuwarden, Elisabeth-TweeSteden hospital, Laurentius hospital, Gelre hospital and Hagaziekenhuis.

### Intervention strategies

#### Transanal minimally invasive surgery (TAMIS)

This procedure was first described by Atallah et al. [11]. The procedure will be performed under either general or spinal anaesthesia at the discretion of the anaesthetist. For this trial only the GelPOINT® path transanal access platform will be used. This is to ensure the greatest possible uniformity in procedures and was already being used by the vast majority of surgeons prior to the study. After insertion of the instruments the margins of the lesion may be marked with coagulation dots to facilitate the incision at the lesion margins at the discretion of the surgeon. The incision must be placed at a distance of at least 5 mm around the border of the lesion to prevent thermal damage complicating the histological assessment. If a lesion is very distal (i.e., at or just above the dentate line), the distal margin can be incised using standard transanal retractors and electrocautery. Before the start of the lateral or proximal portion of the dissection, the TAMIS port can be inserted to be used for the remainder of the dissection. Ideally a partial thickness resection of the lesion will be performed following the intramuscular plane of the muscularis propria using a diathermic hook but a full thickness resection may be performed at the discretion of the surgeon. The wound may be closed, if required, with laparoscopic suture material in a transverse direction so as not to narrow the lumen of the rectum. The type of sedation and precise instruments will be noted in the CRF. Pneumorectum will be achieved using CO2 for insufflation. Initial pressure settings should be between 12- and 20-mmHg and can be increased if there is difficulty in maintaining distension for visualisation. An anal block with bupivacaine or ropivacaine bilaterally is recommended. All other aspects of the procedure and post-procedural care are at the discretion of the operator.

#### Endoscopic submucosal dissection (ESD)

After insertion of the endoscope the margins of the lesion may be marked with coagulation dots to facilitate the incision at the lesion margins at the discretion of the endoscopist. The lesion will be ‘lifted’ by injection of fluid into the submucosa. The choice of injection fluid is at the discretion of the operator. A partial or full circumferential incision will be made around the lesion (at the discretion of the operator) at a distance of at least 5 mm from the border of the lesion to prevent thermal damage complicating the histological assessment. Dissection will take place in the submucosal layer underneath the specimen just above and parallel to the underlying muscularis propria layer. The choice of ESD knife is at the discretion of the operator and must be recorded in the CRF. All procedures will be performed with a high-resolution magnifying video- endoscope. The procedure will be performed under sedation, not general anaesthesia. The choice of sedation technique is at the discretion of the endoscopist but Propofol sedation is recommended. In the case of intraprocedural perforation, this will be treated using clips and desufflation of the peritoneal cavity if required, with an intravenous cannula. In the case of minor bleeding from a small vessel, contact coagulation with the tip of a knife or coagulation with haemostatic forceps will be used for haemostasis. In cases of a severe bleeding from a large vessel or artery, haemostatic forceps will be used for haemostasis. If a pulsating large vessel is exposed within the resection wound, clipping can be performed to prevent delayed bleeding. All of this is considered standard care and should be mentioned in the CRF. All other aspects of the procedure and post-procedural care are at the discretion of the operator.

#### Decision-making regarding patient management

Patients will be discussed at the local multidisciplinary meeting (standard care) and decisions regarding the management of the patient including the need for additional radical surgical resection or other treatment options, will be made there in the normal way in accordance with the current national guidelines.

#### Follow-up

The follow-up consists of a rectoscopy performed 6 and 12 months after the TAMIS/ESD by an endoscopist trained in advanced endoscopic imaging techniques. Three white light pictures, 3 enchanced imaging pictures and preferably a short video should be taken of the scar. In case of no visible recurrence 3 biopsies of the scar should be taken. In case of visible benign recurrence an attempt at an endoscopic resection will be performed, or a re-TAMIS. If recurrence is found at the 12-month rectoscopy this will be resected and further surveillance will be planned for 6 months later.

### Informed consent procedure

Patients meeting all criteria stated above will be informed about the trial at the outpatient clinic by a member of the research team. After written consent is obtained the patient will be allocated to either the TAMIS or the ESD group by computerised randomisation. Subsequently, the patient will be scheduled for therapy at a participating centre.

### Intervention failure: cross-over

If the primary procedure fails, cross-over to the other treatment is possible, but only if the specialist that has to perform the cross-over treatment, deems it feasible.

### Quality assurance

#### Expert panel

An expert panel was established for this trial consisting of five gastroenterologists (JH, JB, LM, AK, BB) and three surgeons (PD, EG, RH). All are specialized in the assessment and treatment of advanced polyps and (early invasive) rectal cancers and perform either the ESD or the TAMIS procedures within this trial. All lesions should be approved by the expert panel before randomisation by review of the endoscopy pictures and video. A lesion is deemed as suitable for participation if a minimum of 2 gastroenterologists and 1 surgeon think the lesion meets the criteria. The expert panel can also be consulted in difficult cases (for example when advanced cross-sectional imaging and endoscopic assessment disagree).

#### Experience requirement

Specialists performing ESD or TAMIS in the TRIASSIC study need to have performed at least 25 procedures. The specialists will be asked to send anonymised procedural data including lesion characteristics, procedure time, complications, histology and follow up (if available) of the latest 15 procedures and an unedited video. To be able to perform procedures in the TRIASSIC study at least 10 of these 15 procedures must have resulted in a R0 resection of lesions > 2 cm in size and must have been performed without complications.

### Histopathological evaluation

Appropriate handling of the resected specimens is critical for accurate histological assessment and will be done as follows (identical to standard care). The resected specimen will be pinned onto a paraffin, rubber or cork sheet so that the normal mucosa surrounding the lesion is evenly flattened and the mucosal surface can be observed. The specimen will then be photographed with a millimetre ruler next to it and fixed in formalin. The specimen should, preferably, be examined by a GI pathologist. The specimen should be photographed, measured and macroscopic appearance described including the lesion, mucosal defects, other abnormalities and the resection margins. The specimen should also be inked. A different colour should be used for the resection plane and the edges of defects. A tangent that touches the focus closes to the horizontal margin is assumed. The first cut is carried out in the direction perpendicular to the tangent. Hereafter the specimen is sectioned into slices parallel to the first cut. Lastly all slices should be embedded in cassettes for histological diagnosis. Incomplete (R1) resection is defined as tumour infiltration of the margins and/or if infiltration cannot be determined because of coagulation artefacts. In case of an adenocarcinoma the high-risk factors will be assessed; grade of tumour budding, invasion depth, differentiation grade, presence of lymphovascular invasion and radicality.

#### Central pathology revision

All resection specimens will be revised centrally by an expert pathologist.

### Outcomes

#### Primary study endpoint (for non-inferiority)

1. Cumulative recurrence rate at follow-up rectoscopy after 12 months, histologically confirmed from resected visible residual disease or, if not present, from biopsies of the scar

#### Secondary study endpoints

2. Radical (R0-) resection rate, defined as dysplasia-free vertical and lateral resection margins at histology
3. To compare the perceived burden of the treatment and quality of life among patients using questionnaires ((EORTC) QLQ-C29 [12], EUROQOL EQ-5D-5L [13], COREFO [14]).
4. Overall complication rate*
5. Surgical referral rate defined as the number of patients that are referred for trans-abdominal surgical management at 12 months
6. Cost effectiveness at 12 months.

#### * complications are defined as follows

- *Intraprocedural peritoneal breach: the condition in which the abdominal cavity is visible from the colorectal lumen during the procedure because of mural tissue defects, that requires* (1) *(prolonged) admission or* (2) *surgery*
- *Intraprocedural bleeding: bleeding that occurs during the procedure that cannot be controlled by standard local haemostasis techniques such as electrocoagulation or clips and that requires* (1) *transfusion or* (2) *termination of the TAMIS or ESD procedure.*
- *Postprocedural bleeding: bleeding within 30 days after the procedure resulting in* (1) *new presentation at the hospital,* (2) *hospital admission, transfusion* (3) *or* (4) *repeated intervention to obtain haemostasis.*
- *Postprocedural bowel perforation: a bowel perforation within 30 days after the procedure that is detected after completion of the procedure during which a peritoneal breach did not occur, diagnosed by abdominal pain with focal guarding and a rise in C-reactive protein and/or fever (T > 38.5 C) in combination with free air in the peritoneal cavity at abdominal CT.*
- *Postprocedural serositis: abdominal pain with focal guarding and/or fever (T > 38.5 C) within 30 days after the procedure, but without signs of perforation (free air at abdominal CT) and in the absence of another infection focus (urinary, pulmonary* etcetera*) that requires (prolonged) admission*

### Sample size calculation

The sample size was calculated for the primary outcome parameter, the cumulative recurrence rate at 12 months. To assess the non-inferiority of the ESD procedure the sample size calculation was based on the assumption that the recurrence rate will be 3% in the ESD group and 6% in the TAMIS group based on a systematic review of the literature specifically for studies performed in the West. If there is a true difference in favour of ESD of 3%, then 166 patients are required to be 80% sure that the upper limit of a one-sided 97.5% confidence interval (or equivalently a 95% two-sided confidence interval) will exclude a difference in favour of the TAMIS of more than 6%. (Software: PASS Version 15 – www.ncss.com). We have chosen a non-inferiority margin of 6% because we believe that this difference in risk of benign recurrence between the intervention group and usual care group is clinically acceptable. To allow for patients lost to follow-up (4%) and patients requiring additional surgical resection due to high risk characteristics (12%) in whom the primary outcome cannot be assessed, a total of 198 patients will be included; 99 patients in each arm. The incidence of large rectal non-pedunculated lesions in the Netherlands is estimated to be between 250 and 500 new cases a year. We estimated that the participation of 15 centres will be required to complete the inclusion period within 3 years. To avoid unnecessary delay, we will start this trial with 5 centres and will extend the number of centres during the course of the trial.

### Ethics

This clinical investigation will be conducted in accordance with the ethical principles that have their origin in the Declaration of Helsinki. This clinical investigation will comply with the practices set out in EN ISO14155:2011. This investigation was approved by the Medical Ethics Committee of the Leiden University Medical Center (NL61603.058.18). The study will be conducted according to the rules on medical research involving human subjects (Medical Research (Human Subjects) Act), in Dutch: Wet Medisch-Wetenschappelijk Onderzoek met mensen (WMO). In addition, approval has been obtained from all of the participating hospitals: Amsterdam University Medical Center (Amsterdam Medical Center and Vrije Universiteit University Medical Center), University Medical Center Utrecht, Erasmus medical Center, IJsselland hospital, Alrijne hospital, Haaglanden Medical Center, Diakonessenhuis, Deventer hospital, Meander Medical Center and the St. Antonius hospital.

### Data-analysis

To assess the non-inferiority of the ESD procedure, the difference between the cumulative recurrence rates at 12 months in both groups will be compared to the non-inferiority margin of 3% using a one-sided Mantel-Haenszel test (with alpha 0.025) to account for stratification factors. For the other variables, normality will firstly be assessed. The secondary endpoints will be compared using the student t-test or Mann-Whitney U test and Chi-square or Fisher’s exact test as appropriate. Multivariate regression will be considered for adjustment for possible confounding if necessary. The analysis will primarily be carried out on an intention-to-treat basis.

### Economic evaluation

The Health Economic Expert responsible for the study will perform the Economic Evaluation. The economic evaluation will consist of a cost-effectiveness analysis from a healthcare perspective (CEA: costs per prevented recurrence) and a cost-utility analysis from a societal perspective (CUA: costs per QALY, estimated using the Dutch tariff for the EuroQol EQ-5D-5L at 0, 6 and 12 months). Both analyses will be trial-based, using patient reports, with a one-year time horizon. Costs will include the index interventions with hospitalisation (estimated from patient charts), subsequent hospital and non-hospital healthcare and productivity during the study follow-up (measured using patient questionnaires at 6 and 12 months). Cost-price analyses will be performed for the TAMIS and ESD procedures, including procedure time, materials and anaesthesia. Other healthcare and societal costs will be valued and discounted according to the Dutch guidelines for economic evaluations [15, 16]. Average costs and patient outcome will be compared according to intention-to-treat, using net-benefit analysis, and using multiple imputation to account for missing data.

## Discussion

In 2010 The European Union published recommendations that colorectal cancer screening should be performed in all member states [17]. CRC screening reveals more large precursor lesions for which local excision may be the optimal treatment. However, the choice as to whether to perform local excision and with which technique is still unclear, especially in Western countries where Endoscopic Submucosal Dissection is being introduced slowly and remains controversial. As stated in the introduction, most lesions that are not overtly cancerous on endoscopic inspection are resected by pEMR in Western countries. The limitations of this technique which only allows sub-optimal histological assessment are illustrated by the TREND study; 3 out of 87 (3%) patients had a carcinoma during follow-up after removal of a pT0 lesion in the piecemeal EMR group, versus none in the *en bloc* TEM group [5]. Malignant recurrence at the removal site of a benign adenoma occurs in approximately 1–2% of cases [18, 19]. A possible explanation is pathological under staging with small areas of invasion being missed in the assessment of the pEMR specimens. A different explanation is remnant adenomatous tissue that progresses into a carcinoma. Surgical resection due to uncertain histology after pEMR of large lesions optically staged as benign occurs in 3.5% of cases [20]. These limitations are encouraging a shift away from pEMR towards *en bloc* resections of large rectal lesions.

However, the *en bloc* resection method of choice is unclear. In 2017 the European Society for Endoscopy recommended that a comparison between ESD and local surgical resection is needed to guide decision making for the optimal approach for the removal of large rectal lesions in Western countries [21]. The reason ESD is compared to TAMIS, instead of TEM, in the TRIASSIC study is because TAMIS provides the benefits of advanced videoscopic transanal excision at a fraction of the cost of TEM [11]. No additional investment is required and the TAMIS port has a shorter shaft length, allowing an increased working angle and more distal dissection compared to the TEM port [22]. There are also suggestions that TAMIS may be less traumatic to the anal sphincter, compared to TEM [23].

For this trial it was decided to include all rectal lesions > 2 cm, both including those clinically staged as benign and early invasive. It could be argued that the advantages of *en bloc* resection only outweigh the disadvantages in early invasive lesions. This is the basis for the Japanese indications for colorectal ESD where a lesion must have at least one high risk feature for early invasion. However, for reasons that are unclear, Western centres seem to be less good at recognising these high risk features with very high rates of “covert” cancer in lesions clinically staged as benign [24, 25]. The likelihood of “covert” cancer is associated with lesion size, site within the colon and lesion morphology. In the rectum all clinically benign lesions > 2 cm have a > 5% chance of harbouring a focus of “covert” cancer, regardless of morphology. Piecemeal EMR is therefore an inappropriate treatment in at least 5% of rectal lesions > 2 cm. We feel that this is unacceptably high and that *en bloc* resection is justified in all rectal lesions > 2 cm.

Similarly, clinical staging of massive invasion (>T1 sm1) is also only accurate in 50% in expert Western centres [25]. In the other 50% local *en bloc* resection would have been sufficient. Cross sectional imaging with MRI or Endoscopic Ultrasound does not improve this [26]. Likewise, determination of the N-status of rectal lesions is problematic. MRI has been shown to have a sensitivity of 94% and a specificity of only 67% [27]. A systematic review of the performance of Endoscopic Ultrasound (EUS) showed a pooled sensitivity of 73.2% and a pooled specificity of 75.8% [28]. The consequence of the poor performance of the preoperative staging methods is that the staging of early stage cancers is increasingly being performed by the histology sample obtained by diagnostic *en bloc* resection. This approach has already been formalized for other early GI cancers such as oesophageal cancers [29] but not yet for rectal cancer. Diagnostic resection allows accurate pathological examination but only if performed *en bloc* and when care is taken to ensure optimal orientation of the specimen.

The TRIASSIC study is the first direct comparison between rectal *en bloc* resection techniques in a randomised setting in a Western population. The TRIASSIC study will increase the current knowledge as to which is the preferred minimally invasive resection method for rectal *en bloc* resections. This is important as the detection rate of large rectal lesions (polyps and early cancers) has greatly increased with the introduction of CRC screening. This study will support the optimal use of healthcare resources in the future.

## Supplementary information

**Additional file 1.**

## Data Availability

The datasets generated and/or analysed during this study are not publicly available due to individual privacy but are available from the corresponding author on reasonable request.

## Abbreviations

TRIASSIC: Multicentre, randomised controlled trial comparing TRansanal minimally InvAsive Surgery (TAMIS) and endoscopic Submucosal dIsseCtion (ESD) for resection of non-pedunculated rectal lesions
TAMIS: Transanal Minimally Invasive Surgery
ESD: Endoscopic Submucosal Dissection
pEMR: Piecemeal Endoscopic Mucosal Resection
TEM: Transanal Endoscopic Microsurgery
EUS: Endoscopic Ultrasonography
COREFO: COloREctal Functional Outcome questionnaire
