# Supplementary material for: Transanal minimally invasive surgery (TAMIS) versus endoscopic submucosal dissection (ESD) for resection of non-pedunculated rectal lesions (TRIASSIC study): study protocol of a European multicenter randomised controlled trial
Source: BMC Gastroenterol. 2020 Jul 13;20:225. doi: 10.1186/s12876-020-01367-z (PMC7359465; doi:10.1186/s12876-020-01367-z)
Supplement: Supplementary file 1 — Additional file 1. [file 12876_2020_1367_MOESM1_ESM.pdf]

Participant Information Sheet (PIS)

TRIASSIC

A multicentre, randomised controlled trial comparing TRansanal minimal InvAsive Surgery (TAMIS) and endoscopic Submucosal dIsseCtion (ESD) for the resection of large rectal polyps.

Introduction

Dear Sir or Madam,

Before asking your permission to join our clinical trial we would like to provide you with some additional information. Note that participating in this trial is completely voluntary. To participate we need your written consent. This form is handed to you because a large polyp was found in your rectum. Please take your time to read this form carefully and feel free to ask any additional questions. The independent expert, named at the end of this form, can also answer remaining questions. Feel free to also discuss a possible participation with your partner, friends or family.

1. General information

This trial is initiated by the Leiden University Medical Center (LUMC) in the Netherlands, and conducted in several other hospitals in the Netherlands. For this clinical trial a total amount of 198 participants is necessary. This trial was approved by the ethics committee of the LUMC.

2. Aim of this study

There are currently two different techniques to remove your polyp in one piece: TAMIS and ESD. these techniques are standard care, but have never been directly compared in a clinical trial. The aim of this study is therefore to compare these two resection techniques. The following subjects will be assessed: The amount of successful resections, the amount of patients with residual tissue during follow-up endoscopy, the amount of complications, patients' experiences and the costs.

3. What does participation mean for me?

Participation will last 12 months.

Assessment of eligibility

First we will determine whether you meet the pre-established criteria. A colonoscopy will be performed (or repeated) to evaluate your polyp, in some cases an additional transanal ultrasound will also be performed. In all cases a MRI of the rectum will be performed.

Treatment

Half of the study participants will be treated with the TAMIS procedure, the other half with ESD. At random the computer will appoint one of these two treatments to a patient.

- Transanal Minimally Invasive Surgery (TAMIS): During this procedure, performed by a surgeon, a flexible rubber tube will be inserted in the anus. Through this tube multiple instruments will be inserted to operate in a way comparable to a laparoscopic procedure without the need for an

abdominal incision. To treat the polyp the submucosal layer and in some of the cases one of the muscular layers will be removed, if that is the case then the wound will be closed with internal sutures.

- Endoscopic Submucosal Dissection (ESD): For this procedure, performed by the gastroenterologist, a flexible endoscope will be used and inserted in the anus. The procedure will start with injection of fluid underneath the polyp. After this so called lifting, the polyp will be separated from the surrounding submucosal layer by the use of a small knife. Both muscular layers will be kept intact. There is no need to close the superficial wound within the rectum.

Both of these procedures have benefits and disadvantages. TAMIS is a relatively new procedure where the surgeons use instruments comparable to laparoscopic surgery. Non-flexible instruments will be used and patients will undergo general anesthesia. More tissue will be removed compared to ESD and patients. ESD is also a relatively new procedure that uses a flexible instrument that allows the gastroenterologist to also look and work backwards. The muscle layers of the colonic wall will not be removed and patients will undergo a lighter form of anesthesia where patients will be able to breathe on their own. Because a direct comparison between these two techniques is lacking, it is still unknown which technique will show the best results in the long run and which technique is least of a burden to patients. We hope to answer these questions with the results from this trial.

Hospital visits, measurements

After both the TAMIS and the ESD procedure it is important to plan a rectoscopy after 6 and 12 months to inspect whether there is any residual tissue. If recurrence is visible this will be removed. Biopsies of the scar will be taken in all cases. Five times during this trial a questionnaire will be sent. The questions are mostly about your complaints or symptoms as result of your polyp and the ESD/TAMIS procedure. Filling in these questionnaires will take you approximately 10 minutes.

Other than standard of care

Removal of the polyp will in both cases be according to current guidelines. Thus, this trial is not an experimental trial. The differences with the standard of care are the following:

- The option between either of the 2 techniques will be determined by the computer
- Eligibility will be assessed before participation
- You will be asked to complete questionnaires

4. What will be expected from you

For this trial to succeed and for your own safety it is important that you complete all the appointments in the hospital. It is also important to reach out to the researcher:

- When you are admitted or receiving treatment in the hospital
- When you suddenly experience health complaints
- If you desire to decline further participation in the trial
- If your contact information changes

5. Possible complications and other negative effects

TAMIS and ESD both have complications that could occur. After removal of the polyp there will be a "wound" in the colonic tissue. From this wound a bleeding could arise or a perforation could occur. You have to contact the researcher immediately when there is blood loss or (severe) abdominal pain. In most of the cases the bleeding can be stopped directly during the initial procedure, limiting total blood loss to a minimum. However, bleeding could also occur after your treatment. Then the gastroenterologist will perform a new colonoscopy and try to control the bleeding. The chance that bleeding occurs is very small in both techniques (approximately 1-2%). A perforation is a hole in the colonic tissue. The chances of a perforations after ESD are approximately 5%. During the ESD procedure a perforation can in most cases be closed directly with a clip. During the TAMIS procedure a perforation is made on purpose which is later closed with sutures. It is extremely rare that recognizing and closing of a perforation is not successful, creating the need for a (laparoscopic) surgical intervention. The chance that this occurs is extremely small (<1%).

6. Possible benefits and risks of participation

It is very important for you to weigh the possible benefits and risks before making the decision to participate.

Benefits: Participating in this trial does not mean your polyp will be treated better. You will however, contribute to the medical field by helping to increase the knowledge of the treatment of preference for rectal polyps.

Risks: Participation does not have any disadvantages. Participation does mean that you will have to answer questionnaires, which takes some time.

7. What to do if you no longer want to participate or withdraw consent

You must decide for yourself whether you would like to participate in this clinical trial. Participation is completely voluntary. If you do not want to participate you will be treated according to the standard treatment in your hospital. If you decide to participate you are allowed to withdraw consent without having to give a reason. The researcher must be made aware of this directly. The data collected up until that moment will be used for the research. If during participation new information arises this will be communicated with you. If this information changes the trial considerably you will be asked to give consent, once again.

8. End of the trial

Your participation stops when:

- All study visits [as mentioned in 4.] are completed
- You decide to stop
- The researcher thinks it is better for you to stop
- The government or ethics committee decides to stop the trial

The entire trial is finished when all participants completed the trial. After processing all of the data, the researcher will inform you about the most important findings. This will happen approximately 5 years after participation.

9. Using and storing your data

For this trial your personal data will be collected, used and stored. Data will include name, adress, date of birth and data conserning your health. The collection, usage and storage of your data is necessary to answer the questions of this trial. We ask consent to use your data for our trial.

Confidentiality of your data

Your data will be coded, to guarantee a certain amount of privacy. Your name and other data that could be used to directly identify you will be omitted. The coded data can only be traced back to you by the use of a key. This key will be stored in the research department of that study site. The data sent to the contractor will only be the coded data. The data used for reports and eventual publications will be coded and can't be traced back to you.

Data access for study monitoring

A few people will be granted access to all of your data. Also the data without coding. This is necessary to monitor whether the study is performed correctly. The following people can be granted access to your data for monitor purposes: The ethics committee, the safety and the monitoring board of this trial, the national/international authorities (such as "Health and Youth Care Inspectorate". They are obligated to keep your data classified. We also ask permission to share your data with these people/organizations stated above.

Period of datastorage

Your data must be stored for 15 years at the study site and 15 years at the contractor.

Storage and use of your data after completion of this trial

After completion of this trial, your data can be of great importance for other research regarding polyps. For this reason your data will be stored for 15 years. It is possible for you to participate in the TRIASSIC trial without giving consent for the usage of your anonymous data for further research.

Withdrawal of consent

You can always withdraw your consent. This applies for this trial but also for the usage of data for future research. Data collected until the time of the withdrawal of consent will still be used for research purposes.

More information about the rights and procession of your data

For general information about the rights about participating in clinical trials you can consult the following website: https://www.rijksoverheid.nl/documenten/brochures/2014/09/01/medisch-wetenschappelijk-onderzoek-algemene-informatie-voor-de-proefpersoon

If you have remaining questions about your rights you can contact the person responsible for the protection of your data. Contact information can be found in appendix A. If you have questions or complaints about the possession of personal data we advise you to firstly contact the research department. For more information on privacy and the statement of the hospital you can visit the website of the LUMC. If you have any remaining questions about your privacy you can also contact

the data protection officer.

Registration for this trial

This trial is also registered in the Dutch Trial Registry (Nederlands Trial Register; NTR). No personal data is on this website. A summary will be shown on the website after completion of the trial. You can find this trial with the following number: NTR7281, or the name: TAMIS vs ESD for the removal of rectal polyps, or the acronym: TRIASSIC.

10. Insurance

The risk of patients participating in this trial is equal to patients receiving standard of care (i.e. undergoing the same procedures without being enrolled in this trial). Therefore, the ethics committee of the LUMC granted dispensation for an additional insurance for test subjects.

11. Informing the general practitioner

Because both procedures are standard of care, we will not inform your general practitioner (GP) about the participation in this clinical trial. Your GP will be notified about the medical treatment you will receive.

12. No financial compensation

There will be no form of financial compensation for study participants.

13. Do you have any remaining questions?

If you have any questions you can contact Prof. dr. J.C.H. Hardwick. For impartial advise you can contact: dr. A. Inderson. He knows a lot about this subject but is not involved in this clinical trial.

If you have any complaints concerning this trial, you can discuss these with the local researcher or your specialist. You can also contact the patient service of the hospital. All contact information is in appendix A: Contact information.

14. Signing the consent form

When you feel like you had sufficient time to think, you will be asked to decide whether you would like to participate in this clinical trial. When you give consent, you will be asked to also sign the corresponding consent form. By giving written consent you declare to have understood the information and agree to participate. Both yourself and the researcher will receive a copy of the signed consent form.

Thank you for your attention.

Kind regards,

Drs. N. Dekkers, coordinating investigator LUMC
Prof. dr. J.C.H. Hardwick, principal investigator LUMC

15. Appendices

A. Contact form

B. Consent form

Appendix A: Contact form

Researcher

Prof. dr. J.C.H. Hardwick, Gastroenterologist, phone number +31715265364

Independent specialist

Dr. A. Inderson, MDL-arts, telefoonnummer 071-5269111

Complaints

For complaints you can visit to the patients service desk in the LUMC (Location H2-11, route 473). You can also fill in a digital form. A digital form is also available on: www.lumc.nl

Data protection

If you have any questions regarding protection of your privacy you can contact our Data protection officer (In Dutch: Functionaris gegevensbescherming [FG]). Contactform can be found at www.lumc.nl

Appendix B: Consent form

Transanal Minimally Invasive Surgery (TAMIS) versus Endoscopic Submucosal Dissection (ESD) for the treatment of large rectum polyps: a randomized trial conducted in different hospitals.

- I have read this information folder. I was able to ask questions. I feel like my questions have been answered. I have had enough time to decide whether I would like to participate.
- I am aware that participation is voluntary. I am also aware that I can decide to stop participating at any point during this trial. For this no reason has to be given.
- I give consent to inform my specialist that I am participating in this trial
- I give consent for the collection, usage and storage of my data to answer the research question.
- I am aware that for quality control of this trial, other individuals are granted access to my data. These individuals are mentioned in this information sheet. I give consent for these individuals to have access to my data.
- I give consent to send me questionnaires about my experiences and quality of life, at six different time points, as mentioned in this folder:

Email address: ________________________

If you prefer to receive the questionnaires on paper:
Adress: ________________________
____
____
____

I give consent: □ **Yes**
□ **No**
To also store my data longer for future studies regarding large polyps.

I would like to participate in this trial.

Name participant:

Signature: Date : __ / __ / __

I delcare to have informed this participant about this trial.

If during the trial new information arises that could influence the consent from the participant, I will dicuss this with him/her.

Name of researcher (Or his/her representative):

Signature: Date: __ / __ / __

<*if applicable*>

Additional information is given by:
Name:
Function:

Signature: Date: __ / __ / __

The participant will receive the information sheet, with the signed consent form.
